# Molecular mechanism of leaf adaxial upward curling caused by *BpPIN3* suppression in *Betula pendula*

**DOI:** 10.3389/fpls.2022.1060228

**Published:** 2022-12-01

**Authors:** Kun Chen, Chang Qu, Xiao-yue Zhang, Wei Wang, Chen-rui Gu, Gui-feng Liu, Qi-bin Yu, Chuan-ping Yang, Jing Jiang

**Affiliations:** ^1^ State Key Laboratory of Tree Genetics and Breeding, Northeast Forestry University, Harbin, China; ^2^ Citrus Research and Education Center, University of Florida, Lake Alfred, FL, United States

**Keywords:** *Betula pendula*, BpPIN3, IAA transport, Adaxial-abaxial, leaf

## Abstract

Leaves are one of the vegetative organs of plants that are essential for plant growth and development. *PIN-FORMED* (*PINs*) gene is an indoleacetic acid (IAA) transporter that plays a critical role in leaf development. To determine the function of *BpPIN3* in leaf polarity formation in *Betula pendula*, the transgenic lines with *BpPIN3* overexpression (OE) and *BpPIN3-*reduced expression (RE) were analyzed using the *Agrobacterium*-mediated method. The RE lines displayed the characteristics of leaf margin adaxial upward curling, with lower expression of *BpPIN3* resulting in greater rolling. Tissue localization of IAA in the auxin *GUS* reporter system proved that auxin in the RE was mainly distributed in the secondary veins, palisade tissues, and epidermal cells in the leaf margin area. The auxin content in the leaf margin area was significantly greater than that in the main vein tissue. The cell density of the palisade tissue and the ratio of palisade tissue to spongy tissue in the curled leaf margin of the RE lines were found to be significantly decreased. RNA-seq analysis revealed that the RE hormone-signaling pathway genes were significantly enriched compared with those of the OE and WT lines; in particular, the auxin response-related genes SAURs (i.e., *SAUR23*, *SAUR24*, *SAUR28*, and *SAUR50*) and *GH3.10* were found to be significantly upregulated. qRT-PCR analysis indicated that *BpPIN3* expression at the leaf margin was significantly lower than that near the main vein in the RE lines. In contrast, the expression levels of SAURs and *GH3.10* were significantly higher than those near the midrib. In conclusion, *BpPIN3* regulates the expression of auxin response-related genes and the polar transport of auxin to change the polar form of the proximal and distal axes of birch leaves.

## Introduction

Leaves are important vegetative organs in plants. Leaf morphogenesis is of great significance to plant growth and development (Ichihashi and Tsukaya, 2015; Chitwood and Sinha, 2016). In plant developmental biology, polarity establishment is the core issue related to organ morphogenesis. To study the mechanism of leaf polarity formation, past studies have attempted to define the polarity of the leaf in a three-dimensional spatial axis according to the relative position relationship between leaf primordium and shoot apical meristem (SAM) (Bowman, 2000). This axis comprises the proximal-distal axis, medial-lateral axis, and adaxial-abaxial axis (Bowman, 2000; Bowman et al., 2002; Hasson et al., 2010). Of these, leaf adaxial-abaxial polarity formation is an important leaf development process that is closely related to important physiological processes such as leaf photosynthesis. Several studies have demonstrated that plant leaf adaxial-abaxial polarity formation involves complex gene regulatory networks, which mainly include adaxial-abaxial polarity genes, adaxial-abaxial boundary-related genes, small RNAs, and plant hormones. These factors regulate the leaf adaxial-abaxial polarity formation through synergistic or antagonistic effects (Eshed et al., 2004; Bonaccorso et al., 2012; Iwasaki et al., 2013; Machida et al., 2015). For example, the *HD-ZIP II* and *HD-ZIP III* subfamily members of the *homeodomain-leucine zipper (HD-ZIP)* transcription factor gene family are involved in the regulation of leaf adaxial surface polarity formation. Among them, *REVOLUTA* (*REV*) gene deletion mutant *rev* of the *HD-ZIP III* gene family in *Arabidopsis thaliana* caused abnormal leaf cell division and leaf edge rolling (McConnell et al., 2001; Otsuga et al., 2001). The double-deletion mutants or triple-deletion mutants of *REV* subfamily genes exhibited obvious defective phenotypes. The *rev/phv* double-deletion mutant causes the abaxialization of the plant and the formation of a trumpet-like structure (Prigge et al., 2005). The *phb/phv/rev* triple mutant caused a defective SAM, which produced only abaxial cotyledons (Emery et al., 2003). Studies have revealed that microRNAs were involved in plant proximal-abaxial polarity formation (Juarez et al., 2004; Chitwood et al., 2009). On the adaxial surface of leaves, miRNAs formed a complex with *ARGONAUTE1 (AGO1)*, which further inhibited the translation of its potential target homeodomain/leucine zipper genes *PHABULOSA (PHB)* and *PHAVOLUTA (PHV)*, which were distributed and expressed in a polar manner in the leaf primordium, thereby regulating the adaxial cell development (Kidner and Martienssen, 2004). In addition, *ARP, KANADI (KAN), AUXIN RESPONSIVE FACTOR (ARF)*, and other family genes also play a key role in the formation of adaxial-abaxial leaf polarity. In cotton (*Gossypium* spp.), leaf development was regulated by the auxin response factor *ARF16-1* through transcriptional regulation of *KNOX2-1*. The heterologous expression of this gene in *Arabidopsis* suggested that its overexpression led to the cracking of leaves and leaf abaxial rolling (He et al., 2021). These observations prove that plant leaf development is a complex process involving the transcription factor family, small RNAs, and several hormone-related genes that affect leaf development by regulating the leaf hormone levels.

Auxin is one of the essential hormones in plants. Auxin regulates plant growth and development by creating concentration differences in different tissues through synthetic regulation or transport regulation (Ludwig-Müller, 2011; Adamowski and Friml, 2015; Kasahara, 2016). Similarly, IAA is essential for leaf polarity formation. Auxin not only regulates cell division and promotes cell elongation but also affects the morphogenesis of individual plants, leaves, and other organs due to its concentration gradient (Ding and Friml, 2010; Ljung, 2013; Robert et al., 2015). The *PIN-FORMED* (*PINs*) family is a gene family that encodes auxin polar transport carrier elements. It plays an important role in the auxin flow between cells and mediating auxin regulation (Wisniewska et al., 2006). Different members of the family genes have different functions. In the *Arabidopsis unhinged-1* (*unh-1*) mutant, the expression of *PIN1* at the leaf margin was inhibited, which modified the auxin content in the leaves, resulting in the narrowing of the leaves and obvious serration at the leaf margin (Pahari et al., 2014). In addition, the protein *PIN3* is sensitive to red light (R) and far-red light (FR). Its location within cells can determine the direction of polar auxin transport, which, in turn, affects the distribution pattern of auxin. This event impacts hypocotyl development (Keuskamp et al., 2010). The *APETALA2* transcription factor *DORNROSCHEN (DRN)* interferes with auxin response and *PIN1* expression, resulting in asymmetric auxin distribution in leaves, which further affects the establishment of adaxial-abaxial polarity in leaves (Chandler et al., 2007). The formation of local auxin concentration maximum and minimum in plants through polar auxin transport (PAT) and the establishment of auxin concentration gradient affect leaf development, with PINs being the key participants in this process. Although a sufficient number of studies have detailed how PINs affect the auxin polarity distribution in plants, there is a paucity of such studies on trees.

Birch (*Betula platyphylla × B. pendula*) is an important broad-leaved, fast-growing tree species in China (Wang et al., 2018). Leaves are essential organs for plant growth and development, as they provide energy through photosynthesis, respiration, and storage of nutrients. The development of leaves is a complex process that involves physiological and biochemical changes (Tsukaya, 2005). In our previous study, we discovered that the transcriptomes of leaves of birch and *Betula pendula ‘Dalecarlica’* differed mainly in their auxin signal transduction genes, such as *BpPINs*, *Aux/IAA, ARFs*, and *GH3s* (Bian et al., 2019). Analysis of the expression characteristics of *BpPINs* family genes in birch indicated that 6 *BpPINs* had high expression in the leaves and that the expression of *BpPINs* was significantly positively correlated with the auxin content. The *BpPINs* family genes were believed to be vital for the birch leaf margin’s morphogenesis (Qu et al., 2020). The expression of *BpPIN3* was the highest in the developing first leaf compared with other leaves, tissues, and organs (Kun et al., 2022). This finding suggested that *BpPIN3* plays an important role in the morphogenesis and development of birch leaves. However, the mechanism by which *BpPIN3* affects the leaf margin development remains unclear.

This study aimed to clarify the function of *BpPIN3* by elucidating the molecular mechanism underlying leaf rolling in *BpPIN3-*reduced expression lines. The leaf palisade tissue (PT) cell density, leaf endogenous IAA content, and tissue localization were determined through leaf anatomical observation. The relationship between *BpPIN3* and IAA was accordingly clarified. Based on the differentially expressed gene (DEG) mining through RNA-seq analysis, the key genes regulating leaf adaxial rolling were identified, and the role of *BpPIN3* in leaf polarity formation was determined. These findings offer guidance for the genetic engineering of birch trees for desired traits.

## Results

### Suppressed *BpPIN3* expression leads to adaxial leaf rolling in birch

**Figure 1:**
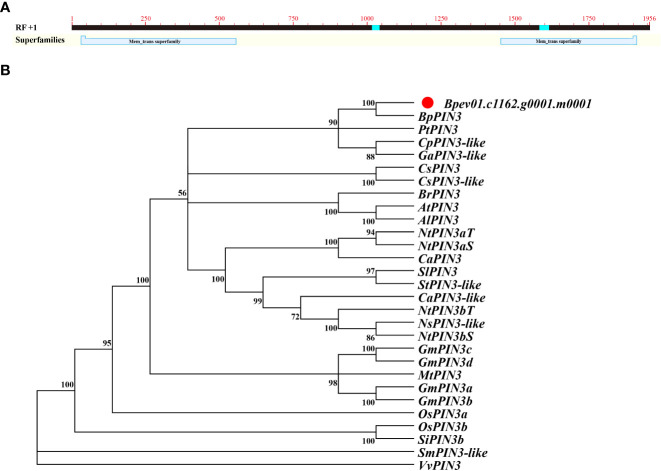
Sequence and phylogenetic analysis of *BpPIN3* **(A)** *BpPIN3* sequence was analyzed by BlastX; **(B)** Cluster analysis was performed on the orthologous sequence of *BpPIN3* and *PIN3* amino acid in different species.

*Bpev01.c1162.g0001.m0001* was identified as the *AtPIN3* homolog with 75.38% similarity in the birch genome. The open reading frame (ORF) of the sequence was 1953 bp, encoding a protein of 650 amino acids (**Figure 1A**). The primers were designed according to the full-length sequence of the ORF and are listed in **Supplementary Table S1**. The cDNA of birch leaves was used as a template for PCR amplification. Gel electrophoresis revealed that the amplified band was visible at 2000 bp (**Figure S1A**). After purification, ligation, and transformation of *E. coli*, single clones were sequenced. The amplified sequence was 1953 bp, which is consistent with the information in the NCBI database.

Protein sequence alignment and phylogenetic tree analysis revealed that the *Bpev01.c1162.g0001.m0001* gene shared high similarity with *PIN3* from other species and 100% similarity with *BpPIN3* from *Betula pendula subsp.pendula*. Therefore, this gene was considered the same as *BpPIN3* of birch (**Figures 1B**; **S1 B**).

*BpPIN3* overexpression lines (OE1–OE3) and the suppressed expression lines (RE1–RE3) were obtained through *Agrobacterium*-mediated zygotic embryo transformation (**Figure S2A**). Total DNA from the leaves of the *BpPIN3* OE lines was used as the template. The 35S:: PIN3 plasmid was used as the positive control, whereas the WT birch DNA was used as the negative control. PCR amplification was performed using two ends of the *BpPIN3* ORF as primers. The results revealed that the positive plasmid and the 3 transgenic lines were amplified in a single band near 2000 bp, which is consistent with the base length of *BpPIN3* sequence (1953 bp). The WT strain showed no amplified band (**Figure S2B**).

The positive target sequence and the complementary sequence were amplified through PCR. The inserted target gene sequence and the reverse complementary sequence in OE lines showed amplified bands, which were consistent with the expected 400-bp length (**Figures S2C, D**). The results indicated that the target gene was integrated into the birch genome.

**Figure 2:**
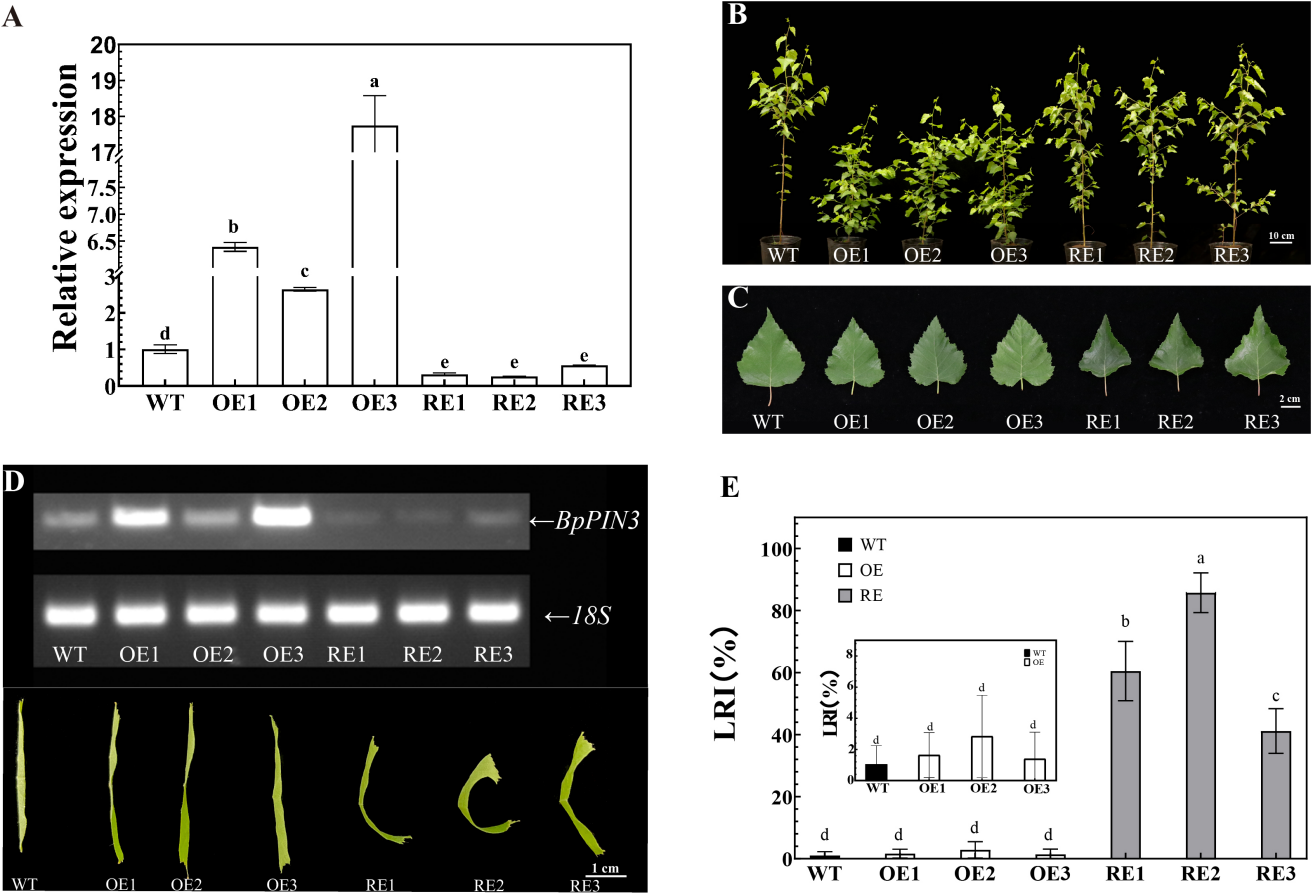
Leaf phenotype of RE, OE, and WT lines **(A)** *BpPIN3* expression in the tested lines. Error bars indicate SD (mean ± SD), n = 3; **(B)** Phenotypic image of 13-month-old test lines, Scale bar = 10 cm; **(C)** Comparison of the third leaf of the tested lines, Scale bar = 2 cm; **(D)** Semi-quantitative RT-PCR electrophoretogram of the tested lines and the curly leaf phenotypes in different *BpPIN3* transgenic lines; **(E)** LRI comparison of the fourth leaf of the tested lines. Error bars indicate SD (mean ± SD), n = 30. Different letters denote statistically significant differences after one-way ANOVA.

The gene expression of *BpPIN3* in three OE lines was significantly higher than that in WT (P< 0.05), as determined through qRT-PCR. The expression of *BpPIN3* in OE3 was 17.74 times higher than that in WT. The expression of *BpPIN3* in the 3 RE lines was significantly lower than that in WT (P< 0.05). The expression of *BpPIN3* in RE2 was the lowest and 3.85 times lower than that in WT (**Figure 2A**). At the same time, qRT-PCR detection of the remaining 5 *BpPINs* in the RE lines revealed that the expression of only *BpPIN3* in the 3 RE lines was significantly lower than that in the WT lines, while those of the other 5 *BpPINs* were significantly upregulated or showed no significant difference (**Figure S3**).

Leaves and seedling height of *BpPIN3* transgenic birch were observed and measured. The leaf length, leaf width, and leaf area of the RE and OE lines were smaller than those of the WT lines, although the differences were nonsignificant (**Supplementary Table S2**). Although OE1-2 was significantly lower than that in the other lines, there was no significant difference in the plant height among OE3, RE, and WT lines (**Supplementary Table S3**). The leaves of RE lines displayed a phenomenon of adaxial upward curling in which the leaf margin curled upward (**Figures 2B, C**). The relationship between the expression level of *BpPIN3* and the degree of leaf rolling was found to be negative (**Figure 2D**). The leaf rolling index (LRI) is an important index that reflects the degree of leaf rolling. Therefore, the LRI statistics indicated no significant difference between the OE lines and WT lines, and the mean values of the 3 lines were all<4%. The LRI of 3 RE lines was significantly higher than that of WT (P< 0.0001), and the LRI of RE2 lines was 85.7% (**Figure 2E**). The decrease in *BpPIN3* expression caused adaxial leaf rolling, whereas the increase in *BpPIN3* expression showed no significant effect on the leaf phenotype.

### Adaxial rolling of *BpPIN3*-RNAi lines caused by the change in palisade cells density

**Figure 3:**
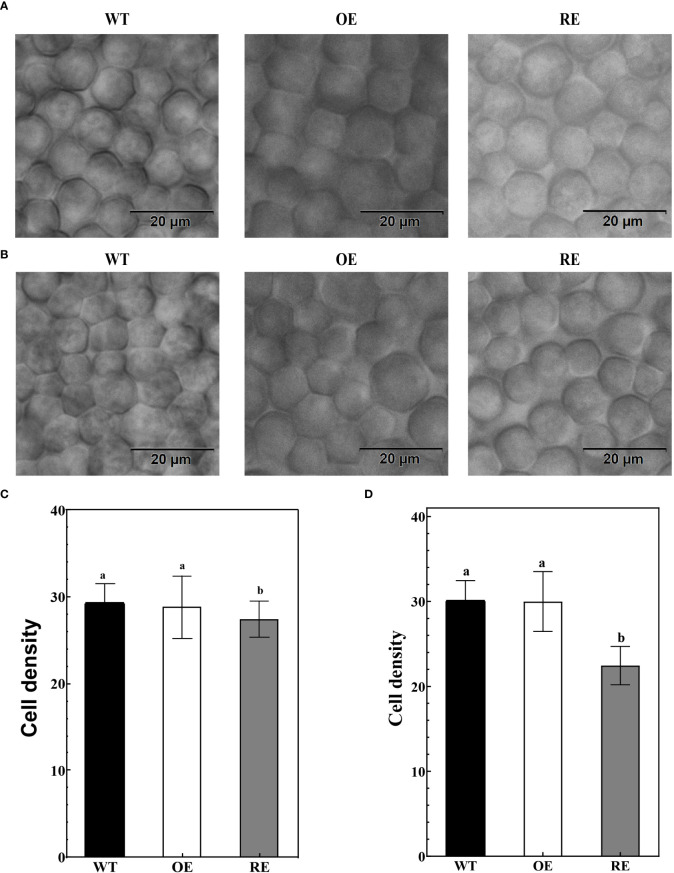
Leaf palisade tissues of the RE, OE, and WT lines **(A)** Observation of the palisade tissue cells in the main vein area; **(B)** Leaf margin palisade tissue cell observation; **(C)** The number of cells per unit area of the palisade tissues in the main vein area; Error bars indicate SD, n = 70; **(D)** Number of cells per unit area of the leaf margin palisade tissues. Error bars indicate SD, n = 70. Different letters denote statistically significant differences after one-way ANOVA.

After transparentizing the mature leaves of the tested lines, palisade cells density showed no statistically significant difference between the OE lines and WT lines (**Figures 3A–D**). However, the palisade cells density of the RE lines was significantly lower than that of the WT and OE lines (P< 0.0001). The palisade cells density in the main vein of the RE lines was 27.42 (cells/µm^2^), which was 6.16% and 4.96% lower than that of the WT and OE lines, respectively (**Figure 3C**). The density of palisade cells in the leaf margin area was only 22.44 (cells/µm^2^), which was 25.47% lower than that of WT lines and 25.18% lower than that of the OE lines (**Figure 3D**). This finding indicated that compared with WT and OE lines, the palisade tissue cells of RE lines are loosely arranged. The closer to the leaf margin, the more obvious the degree of looseness.

**Figure 4:**
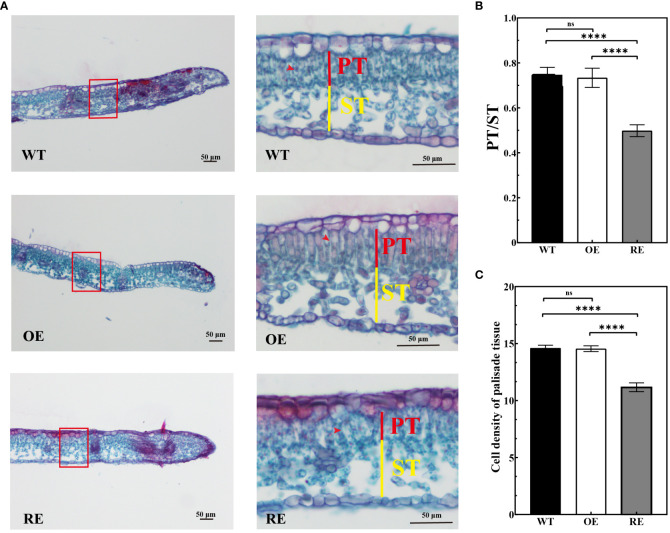
Leaf margin section observation of the RE, OE, and WT lines **(A)** Leaf margin sections of RE, WT, and OE lines, Scale bar = 50 μm; **(B)** Marginal zone ratio of the test lines; **(C)** Cell density of palisade tissues under the transverse view of the leaf margin area. Error bars indicate SD, n = 30. Data were analyzed by one-way ANOVA. ****p< 0.0001 and ns, no significance.

The anatomical observation indicated no significant difference between the OE lines and WT lines in terms of the palisade/spongy ratio and PT density at the leaf margin curling site. However, the palisade/spongy ratio and PT density of the RE lines were 33.33% and 30.1% lower, respectively, than those of the WT lines (**Figures 4A–C**).

To determine whether the morphology of the leaves of the RE lines was changed after flattening, 13 landmarks (**Figure S4A**) were marked on the leaves of the tested lines, and the original coordinate matrix was transformed into a covariance matrix (**Figure S4B**) by using Procrustes of MorphoJ. The results of principal component analysis (PCA) revealed that the principal components PC1 and PC2 accounted for only 47.33% of all leaf type differences, whereas PC1 (28.78%) could not significantly distinguish each strain (**Figures S4C, D**). This finding indicated that the leaf type of RE, OE, and WT in the flat state did not change significantly. The adaxial rolling of the leaves of RE lines did not induce changes in their leaf type.

### Differential expression of IAA responsive genes in *BpPIN3*-RNAi lines

**Figure 5:**
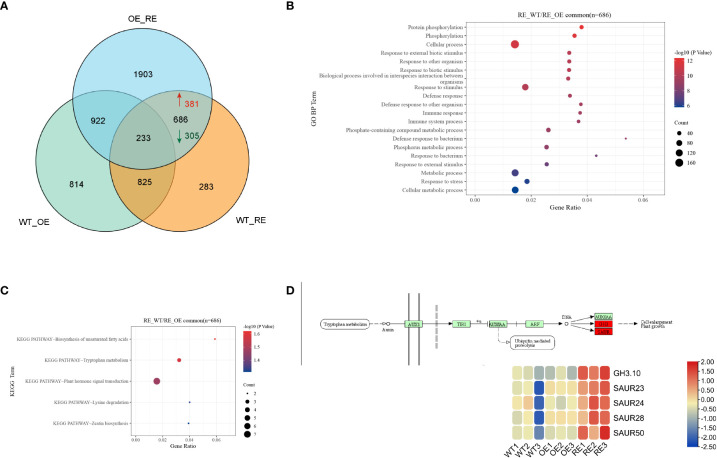
Transcriptome sequencing analysis of the RE, OE, and WT lines **(A)** Veen diagram of DEGs among RE, OE, and WT lines; **(B)** Target interval GO enrichment analysis; **(C)** Target interval KEGG enrichment analysis; **(D)** Heat map of gene expressions in the auxin signaling pathway.

To identify the DEGs in *BpPIN3* transgenic birch, RNA-seq analysis was performed on mature leaves of the RE, OE, and WT line, and the differentially expressed genes (DEGs) were analyzed based on the following criteria: p< 0.05 and|Log_2_Foldchange| ≥ 1. PCA of the transcriptome data clearly displayed differences among all transgenic lines. Each point represents the transcriptome of a different sample (**Figure S5**). As the leaf shape of OE and WT lines is the same, the analysis only focuses on the related DEGs in WT-RE and OE-RE. The results revealed that there were 686 DEGs in the intersection, of which 381 DEGs were upregulated and 305 DEGs were downregulated in the RE lines when compared with WT lines (**Figure 5A**). GO enrichment of the DEGs in the intersection displayed significant enrichment pathways mainly in the cell surface receptor signaling pathway, plant organ development, regulation of the hormone levels, and response to jasmonic acid (**Figures 5B**; **S6**). Through KEGG enrichment analysis of the intersection, 686 DEGs were found to be enriched in 5 pathways including unsaturated fatty acid biosynthesis, tryptophan metabolism, hormone signal transduction, lysine degradation, and zeatin biosynthesis (**Figure 5C**). Further analysis of the 5 enriched KEGG pathways revealed that the hormone signal transduction pathway was mainly related to the auxin signal pathway, and the 5 genes including *GH3.10* and SAURs in this pathway displayed an upregulated expression trend in the RE lines (**Figure 5D**). This finding indicated that auxin response-related genes were significantly highly expressed in the RE lines. The expression of these 5 DEGs was verified by quantitative real-time reverse transcription PCR (qRT-PCR) analysis, which was consistent with the DEG data (**Figure S7**).

### Low expression of *BpPIN3* promotes IAA accumulation in birch leaves

**Figure 6:**
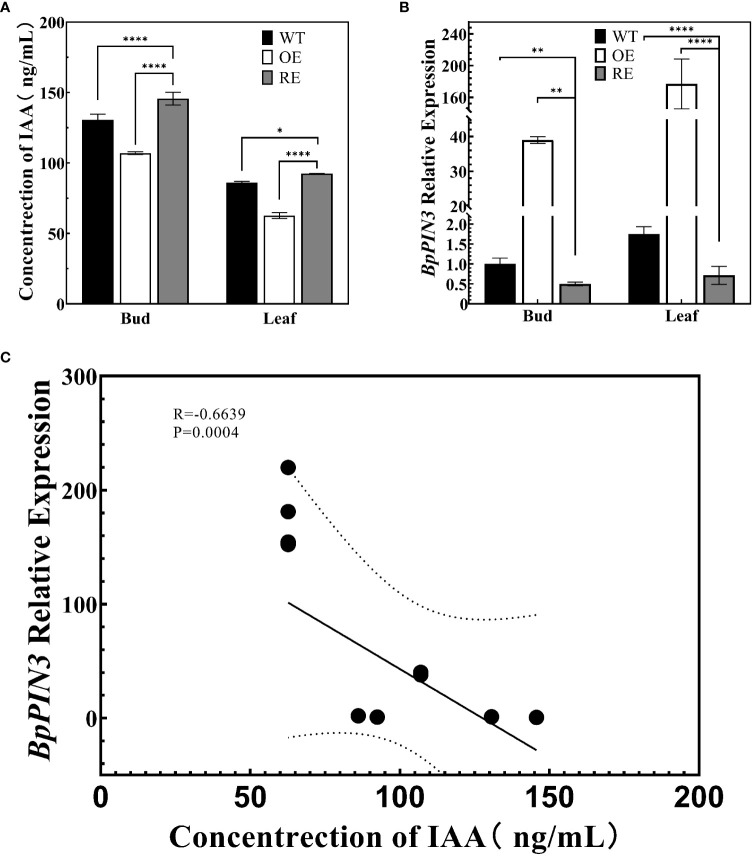
Auxin content and *BpPIN3* expression in apical buds and leaves of the tested lines **(A)** Auxin content in the shoot apex and leaves of the RE, OE, and WT lines. **(B)** Relative expression of *BpPIN3* in the shoot apex and leaves of the RE, OE, and WT lines. **(C)** Pearson’s correlation analysis of auxin and *BpPIN3* expression. Data were analyzed by one-way ANOVA. Error bars indicate SD, n = 3. *p< 0.05; **p< 0.01; ****p< 0.0001.

RNA-seq analysis revealed that the expression of IAA response-related genes in the leaves of RE lines increased significantly. To determine whether the increased expression of these genes affected the auxin content in the RE lines, the IAA contents in the top buds and the first leaves of RE, OE, and WT lines were measured through high-performance liquid chromatography-mass spectrometry (ESI-HPLC-MS/MS) (**Figure 6A**). The results indicated that the IAA content of the RE lines was significantly higher than that of the WT lines, whereas that of the OE lines was significantly lower than that of the WT lines (P< 0.0001). The expression of *BpPIN3* in the top buds and the first leaves of the RE lines was significantly lower than that of the WT lines, while it was significantly higher in the OE lines than in the WT lines (**Figure 6B**). The correlation analysis between the expression of *BpPIN3* and IAA content demonstrated that the expression of *BpPIN3* in the RE, OE, and WT lines was significantly negatively correlated with the IAA content (P = 0.0004) (**Figure 6C**).

### Asymmetric distribution of auxin in the leaves of *BpPIN3*-RNAi lines leads to leaf rolling

**Figure 7:**
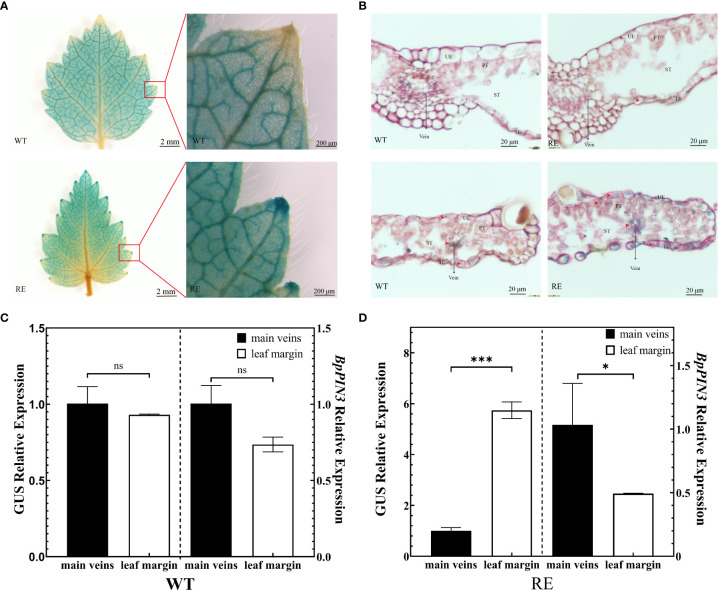
Asymmetric distribution of auxin in the leaves from the RE lines **(A)** *GUS* staining of the leaves from the RE and WT lines, Scale bar = 2 mm and enlarged at the leaf margin, Scale bar = 200 μm; **(B)** Slice observation after *GUS* staining in the main vein region and leaf margin region, Scale bar = 20 μm; **(C)** *GUS* and *BpPIN3* expressions in the WT lines; **(D)** *GUS* and *BpPIN3* expressions in the RE lines. Error bars indicate SD, n = 3. Data were analyzed by Tukey’s tests. *p< 0.05; ***p< 0.001. ns, not significantly different.

To clarify whether the change in the auxin content causes leaf rolling in the RE lines, the IAA reporter vector proDR5:: *GUS* was introduced into the genome of RE2 lines as the receptor, resulting in the RE (proDR5:: *GUS*) lines. The WT (proDR5:: *GUS*) line was set as the control. The auxin signal was visualized by *GUS* staining of transgenic lines to understand the distribution of IAA in the leaves of the RE lines (**Figure 7A**). The observation of the stained leaves revealed that *GUS* staining was almost negligible in the leaf tips of the WT lines. *GUS* staining of the leaf tip of the RE strain was more obvious than that of the WT (**Figure 7B**). The tissue section observation revealed that the *GUS* staining sites of the WT strain were mainly concentrated in the main vein and the secondary vein of the leaf margin, and the color was uniform. The *GUS* staining of the RE lines was lighter in the main vein area, whereas, in the leaf margin area, it was deeper than that in the main vein area. *GUS* staining was also clearly visible in the epidermal cells and PTs at the leaf tip. Since *GUS* staining represents the distribution of IAA, the IAA content in the secondary veins of the leaf margin of the RE lines was significantly higher than that in the main veins, epidermal cells, and PTs containing IAA. No significant difference was noted in the IAA content between the main vein and the secondary vein of the WT lines, and there was no IAA distribution in the epidermal cells of the leaf tip.

The asymmetric distribution of auxin in plants is caused by the polar transport of auxin by *PINs*. To understand the relationship between the asymmetric distribution of IAA content and the expression of *BpPIN3* in leaves, the leaves in the main vein region and leaf margin region of the RE (proDR5:: *GUS*) and WT (proDR5:: *GUS*) lines were used as materials to determine the *GUS* enzyme activity. The relative expression levels of *GUS* and *BpPIN3* were analyzed by qRT-PCR. The results revealed no significant change in the *GUS* activity in the leaf margin and main vein region of the WT lines; however, *GUS* activity in the leaf margin of the RE lines was significantly higher than that in the main vein region (**Figure S8**). *GUS* and *BpPIN3* expressions in the main vein and leaf margin of the RE lines were significantly different (P< 0.05) (**Figures 7C, D**). However, no significant difference was noted between the WT lines (**Figures 7C, D**). The correlation analysis revealed a significant negative correlation between *BpPIN3* and *GUS* expression (P< 0.0001) (**Figure S9**), implying that the decrease in *BpPIN3* expression in the leaf margin of the RE lines may be the reason for the increase in IAA content.

RNA-seq revealed the expression levels of auxin response genes, *GH3.10* and *SAURs*, in the auxin signaling pathway that was enriched by the significantly upregulated DEGs between the RE lines and OE and WT lines. These genes are known to be directly related to the auxin content, and an increase in their expression can increase the auxin content to a certain extent. To further explore whether these genes are related to the change in auxin content in the leaf margin of the RE lines, the expression levels of these genes in different parts of the RE lines were determined. The expression levels of each gene in the leaf margin of the RE lines were found to be higher than those in the main vein area (**Figure S10**), which possibly led to an increase in the auxin content in the leaf margin, consistent with the results of *GUS* staining.

## Discussion

### *BpPIN3* is involved in the establishment of adaxial-abaxial polarity in birch leaves

The morphogenesis of normal leaves requires the establishment and coordination of the polarity of the adaxial-abaxial, adaxial-lateral, and basal-apical axes (Li et al., 2005; Liu et al., 2011). Abnormalities in the development of adaxial-abaxial polarity can cause an upward or downward rolling phenotype of the leaves (Moon and Hake, 2011). Some key genes that impact leaf rolling by controlling the development of leaf adaxial-abaxial polarity have been discovered. *SHALLOT-LIKE1*(*sll1*) mutation in rice (*Oryza sativa*) causes leaves to roll up along the adaxial side by reducing the number of vesicular cells in that specific area. Conversely, the overexpression of *SLL1* leads to a reduction in the vesicular and sclerenchyma cells, resulting in leaf rolling (Zhang et al., 2009; Xiang et al., 2012). *AS1* deficiency inhibits the formation of initial cells in the lateral organs, causing leaf rolling (Byrne et al., 2000). *ASYMMETRIC LEAVES2 (AS2)* inhibits the transcription of the abaxial-determinant genes *ETTIN/ARF3, KANADI2*, and *YABBY5* and affects the establishment of adaxial-abaxial polarity, resulting in leaf rolling (Kojima et al., 2011). The leaf-rolling phenotype is regulated by one or more genes, often requiring synergy with the auxin transport carrier-related genes (Hibara et al., 2009; Zhang et al., 2009). Among them, PINs are involved in auxin polar transport and play a crucial role in leaf development (Scarpella et al., 2006; Chandler et al., 2007; Pahari et al., 2014). In *Arabidopsis*, leaf flattening requires the polar auxin transport. *PIN* inhibition led to flattening defects (Legris et al., 2021). Nevertheless, *PIN3*, which is a member of the PIN family, has rarely been reported to be involved in the establishment of leaf adaxial-abaxial polarity. The leaves of the *mtpin1/mtpin3* double mutant displayed an abnormal abaxial surface only in *Medicago truncatula* (Zhang et al., 2020). In our study, *BpPIN3* suppressed expression transgenic lines (RE lines) showed leaf rolling; however, this phenotype was distinct from that of the *mtpin1/mtpin3* double mutant in that the adaxial showed an upward rolling (**Figure 2C**). This observation may be attributed to the different leaf structure of *M. truncatula* and birch, and the different effects of *PIN3*. Moreover, we found that the expression of the remaining 5 *BpPINs* in the RE lines did not decrease significantly (**Figure S3**). The expression of *BpPIN3* exhibited a negative relationship with the LRI values in the leaves of the RE lines (**Figure 2C**). We, accordingly, speculated that the phenotype of leaf adaxial upward curling was caused by the inhibition of *BpPIN3* in the RE lines. Further study is thus warranted to determine the effect of *BpPIN3* on the polarity development in birch leaves.

### The normal arrangement of PTs is essential for maintaining the polarity of leaves

Auxin is an important plant hormone, and its precise distribution is crucial for the regulation of local auxin concentration and the formation of an auxin concentration gradient in plants. This auxin distribution pattern is mainly regulated by *PINs* (Adamowski and Friml, 2015). The auxin concentration in different parts of the leaves is controlled by plants through PAT, which control the leaf morphology (Reinhardt et al., 2003). Asymmetric polarity of auxin can influence the development of leaf polarity and may lead to the development of a leaf-rolling phenotype in plants (Bowman et al., 2002; Liu et al., 2011). In this study, the auxin *GUS* reporter system was used to analyze the auxin distribution in the RE lines. It was found that auxin was mainly distributed in the epidermal cells and PTs of the leaf margin (**Figures 7C, D**; **S8**). In *Arabidopsis*, auxin was synthesized and aggregated at the leaf margin during leaf development and further transported by the auxin transport carrier PINs from the convergence point to the secondary veins, mesophyll, and other parts of the leaf, eventually triggering cell proliferation and vascular bundle formation for the complete leaf development (Scarpella et al., 2006). During this transport process, the change in auxin concentration affected the development of PTs and spongy tissues of the leaves and then regulated the polarity formation of the adaxial-abaxial axis. The *icu6/AXR3* mutant of *Arabidopsis* demonstrated an abnormal accumulation of auxin in the PTs at the edge of leaves, which decreased the cell size and thus the curling of the adaxial surface of leaves (Pérez-Pérez et al., 2010). Our study revealed that the number of cells per unit area of PTs and the ratio of PTs to spongy tissues in the RE lines decreased significantly in the curled portion of the leaf margin, whereas no significant changes were observed in the OE lines (**Figures 3D**; **4B, C**). Based on the loose arrangement of PTs in the leaf margin of the RE lines, the normal arrangement of PTs seems extremely important to maintain the polarity of leaves. We, accordingly, speculated that the normal expression of *BpPIN3* is beneficial to maintain the normal arrangement of PTs of birch.

### *BpPIN3* and auxin responsive genes regulate leaf polarity formation

The establishment of the adaxial-abaxial polarity of plant leaves is a complex process regulated by interactions among genes, small RNAs, and plant hormones (Moon and Hake, 2011). In this study, 5 DEGs were significantly enriched in the hormone signal transduction pathway by RNA-seq analysis, all of which were significantly upregulated. These enriched genes included *GH3.10* and 4 SAURs genes (i.e., *SAUR23, SAUR 24, SAUR 28*, and *SAUR 50*). The expression of these genes in the leaf margin tissues of the RE lines was significantly higher than that in the middle vein tissue (**Figure S10**), whereas the expression of *BpPIN3* showed the opposite trend (**Figure 7C**). As an important auxin transport carrier, *PINs* respond to external stimuli to form a dynamic and strictly controlled auxin gradient in plants. The synergy of feedback mechanisms requires the rapid removal of auxin from cells when the auxin gradient is established (Friml et al., 2002; Woodward and Bartel, 2005). This process is mainly affected by *GH3s* (Vieten et al., 2005; Hayashi et al., 2021). SAUR proteins influence plant growth and development by altering auxin transport to adjust the auxin levels (Kant and Rothstein, 2009; Chae et al., 2012). In *Arabidopsis*, the overexpression of *AtSAUR19* increased auxin transport in hypocotyls, whereas the knockout of *AtSAUR19* decreased auxin transport (Spartz et al., 2012). A similar phenomenon was observed in *AtSAUR41/63* (Chae et al., 2012; Spartz et al., 2012; Kong et al., 2013). Increasing IAA content in the leaves promoted the expression of *GH3.5* (*WES*), resulting in the rolling of the abaxial surface of leaves in *Arabidopsis* (Park et al., 2007). Increasing free auxin in *Arabidopsis* dominant mutant yucca leads to increasing IAA inducible gene expressions, such as IAA/AUX, SAURs, and GH3s (Zhao et al., 2001). The high concentration of IAA promotes the rapid expression of *GH3* and SAURs. In this study, the auxin content in the leaf margins of the RE lines was higher than that in the main veins, and SAURs were significantly upregulated at the leaf margin. The increase in auxin content may be attributed to the increase in *SAURs* expression in the leaf margin, and the increase of auxin content leads to the rapid induction of GH3.10, which leads to the amino acid reaction of IAA. As the expression of *BpPIN3* in the leaf margin of the RE lines was significantly reduced, the normal IAA transport could not be completed, eventually leading to the formation of an auxin gradient between the leaf margin and the main veins of the RE lines.

**Figure 8:**
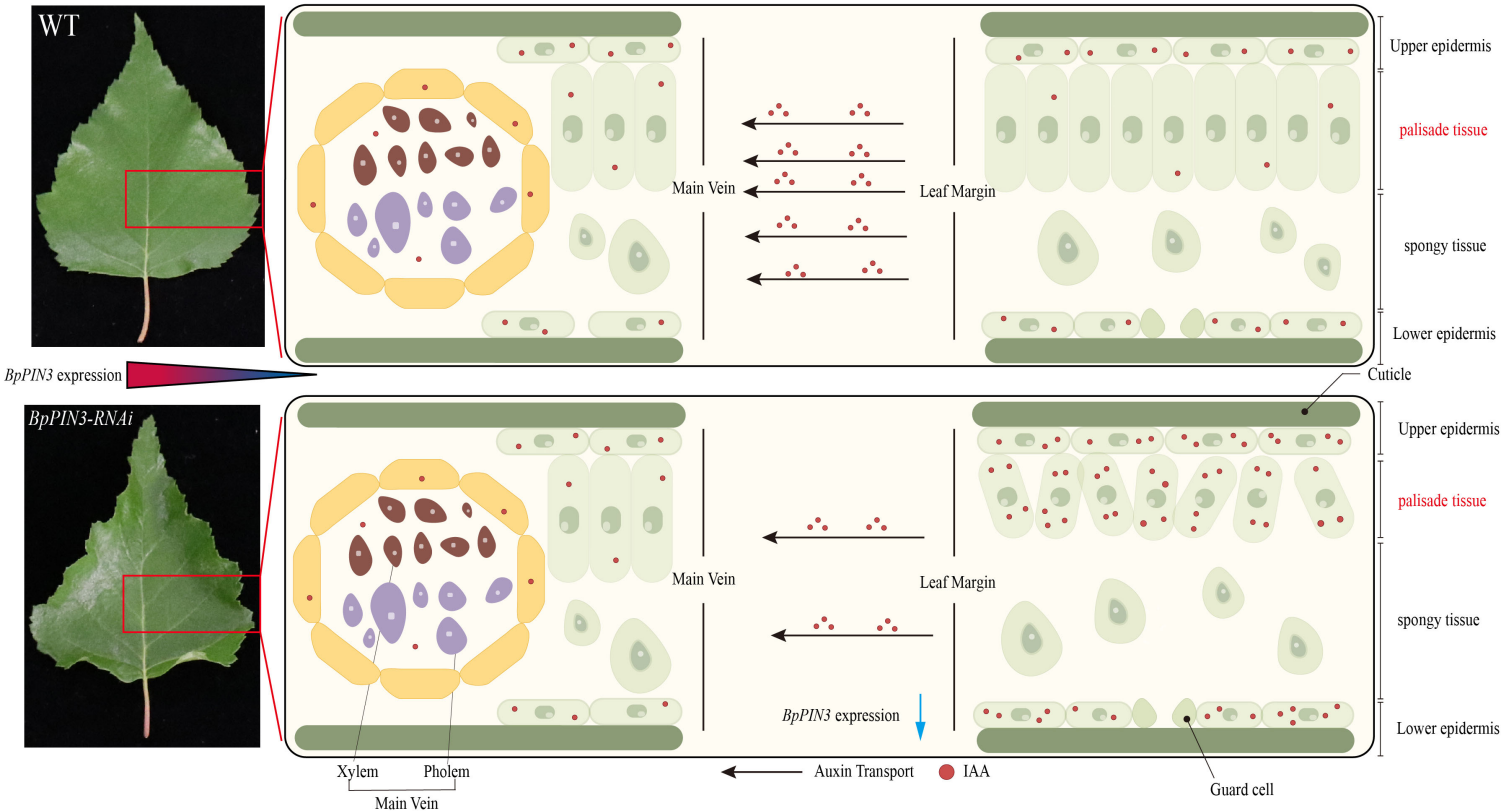
The schematic diagram of *BpPIN3* regulating a leaf adaxial upward curl. Blue arrows indicate the decreased content or function.

In conclusion, *BpPIN3* regulates the auxin response-related gene expression and auxin polar transport in birch leaves, thereby regulating the polarity formation of the proximal and distal axes of the leaves (**Figure 8**). *BpPIN3* expression was significantly downregulated, and the expression of *BpSAURs* was significantly upregulated in the leaf margin tissue cells of the *BpPIN3* RE lines. This event promoted the increase in IAA content and subsequently induced the expression of *BpGH3.10*. Although *BpGH3.10* is involved in the synthesis of free IAA amino acid. However, only a part of IAA formed IAA-Glu and IAA-Asp amino acids, and most of IAA was retained in the upper and lower epidermal cells and secondary veins (**Figures 7C**; **S10**). Because the expression level of *BpPIN3* in the leaf margin tissue cells of the RE lines was significantly lower than that in the middle vein, the accumulated IAA in this area could not be transported to the other parts of the leaf in a timely manner. Consequently, the asymmetric distribution of the IAA content in the leaf margin area was found to be significantly higher than that in the main vein area. Meanwhile, the density of PT cells was reduced and the arrangement was loose in the leaf margin area. The adaxial surface of the leaf was found to be curled.

## Materials and methods

### Plant material

*BpPIN3* gene was cloned from birch, and the mature seeds of birch were used as transgenic plant material. The seeds were collected from a self-pollinated plus tree at Birch Suk improved variety base at the Northeast Forestry University (126°64’E, 45°72’N). After the bracts and periodicals were removed and sealed, the dried, mature inflorescences of birch were stored in a refrigerator at −20°C.

The main vectors included pPROKII and pFGC5941, and the strains were EHA105 and EHA105 (proDR5:: *GUS*). The strains were preserved in our laboratory.

### Cloning and sequence analysis of *BpPIN3*

The mRNA sequence of *PIN3* of birch was obtained by inputting the GenBank login MG198788.1 of *BpPIN3* in the NCBI (https://www.ncbi.nlm.nih.gov/). The upstream and downstream primers were designed according to the mRNA sequence. cDNA of birch leaves was used as the template for PCR amplification. The PCR product was purified and ligated to the pCloneEZ vector to create a recombinant plasmid. After transformation, the monoclonal was picked and sequenced by Beijing Qingke Biotechnology Co., Ltd. The ORF and conserved domain of *BpPIN3* were determined using the ORF search and the conserved domain search service tools of NCBI, respectively. Based on the BlastX alignment, NCBI was searched for protein sequences similar to *BpPIN3.*

The orthologous genes of *BpPIN3* were searched on NCBI, and a Neighbor-Joining phylogenetic tree was constructed using ClustalX 1.83 and MEGA7.0.

### Construction of *BpPIN3* plant expression vector and genetic transformation

The overexpression vector was constructed according to the ORF sequence of *BpPIN3*. Upstream and downstream primers were designed to contain *BamHI* and *KpnI* restriction sites, respectively (**Supplementary Table S1**). The cDNA isolated from the birch leaves was used as a template for PCR amplification. The PCR product and pPROKII plant expression vector were digested with *BamHI* and *KpnI*, respectively. The PCR product and pPROKII plant expression vector were ligated with T4 ligase overnight at 16°C. The positive recombinant plasmid (35S:: PIN3) was obtained by screening on a 50 mg/L Kan resistance plate. The desired sequence was obtained after sequencing, and the positive recombinant plasmid was transformed into *Agrobacterium* EHA105 through electroporation (Huang et al., 2014).

Suppression expression vector was constructed using the NCBI Conserved Domains tool, which predicted 200-bp specific sequences of *BpPIN3*. These sequences were used as the target sequence in the RNAi vector to design forward and reverse primers (**Supplementary Table S4**). The forward and reverse fragments were assembled into pFGC5941 with restriction enzymes and through ligation (Li et al., 2021). Electroporation of *Agrobacterium* EHA105 yielded bacteria engineered for EHA105 (PIN3-RNAi). The transgenic birch plants were obtained *via Agrobacterium*- mediated zygotic embryo transformation (Huang et al., 2014).

The culture medium formula is depicted in **Supplementary Table S1**. The temperature in a tissue culture room was maintained at 25 ± 2°C, with a 16/8-h photoperiod and a light intensity of 1000–1500 lx.

Transgenic birch and wild-type birch (WT) lines were used to extract DNA using the DNAquick Plant System (TIANGEN). The PIN3-RNAi and 35S:: PIN3 plasmid were used as the positive control, whereas the WT line DNA was used as the negative control. PCR detection of transgenic lines was performed with specific primers listed in **Supplementary Table S5** (Huang et al., 2014).

### qRT-PCR detection of transgenic birch

The transplantation of transgenic and WT birch tissue culture root seedlings was performed in a 25-hole seedling tray measuring 40 × 40 cm^2^. This procedure was performed in late May after the seedlings were cultured in a soil matrix medium. The plants included *BpPIN3*-reduced expression lines (RE1-RE3), overexpression lines (OE1-OE3), and the WT birch. In total, there were more than 100 transplanted plants. In the beginning of May of the second year, 30 seedlings showing the same growth vigor from each line were selected and transplanted into a 28 × 28 cm^2^ seedling pot. A mixture of peat, black soil, and vermiculite in a ratio of 3:2:1 was used as the substrate. All flowerpots were filled with the same amount of substrate and placed in a plastic greenhouse under the same management practice in the Birch Seed Base of the Northeast Forestry University.

Total RNA was extracted from the test lines by using the RNA extraction kit (RNeasy Plant Mini Kit, Biotake) and then reverse transcribed into cDNA by using the ReverTreAce^®^ qPCR RT Kit (Toyobo, Osaka, Japan). SYBR Green PCR master mix (Toyobo) was used to perform real-time quantitative PCR (qRT-PCR) on the ABI 7500 Real-Time PCR system. *Bp18S* rRNA was used as an appropriate internal reference gene, and gene-specific primers were designed using Oligo7 software (**Supplementary Table S6**). The qPCR results were analyzed using the 2^-ΔΔCT^ method, and each reaction was repeated thrice (Huang et al., 2014).

### Leaf morphological characteristic determination

The healthy leaves of plants from the RE, OE, and WT lines were selected, and 20–30 plants were selected from each line. The highly mature leaves (fourth leaves) of each plant were selected. The leaf images were captured using a scanner and analyzed using ImageJ software. This allowed for the measurement of leaf length (Ll), leaf width (Lw), natural leaf margin distance (Ln), leaf perimeter (LP), and leaf area (La). The ratios Ll/petiole length (Pl) and Ll/Lw, as well as the LRI, were also calculated.


$$LRI\;=\;\frac{\mathrm{(L}w-L\mathrm{n)}}{Lw}\;\times \;100\%$$

where Lw is the widest width of the leaf at full extension and distance, and Ln is the distance between the leaf margins under natural conditions. Cell density determination of leaf PTs

The mature leaves (the fourth leaf) of the RE, OE, and WT lines were vacuum-fixed in the FAA solution for 48 h and then placed in a chloral hydrate solution (chloral hydrate 200 g; glycerin 20 g; water, 50 mL). Palisade cells near the main vein and the leaf margin were observed under an optical microscope (OLYMPUS BX43F). Cell density was measured using image analysis software (Image J) (Schneider et al., 2012). Each measurement was repeated thrice, and the leaves of three individuals of each line were used. The number of cells in the four fields of view was measured for each part. The cell density was the number of cells in a unit area (50 μm^2^).

### Leaf anatomical observation

For the anatomical observation, mature leaves (fourth leaves) from the RE, OE, and WT lines were selected. The leaves with the widest leaf margins of 1 × 1 cm^2^ were sliced using the conventional paraffin section method (Liu et al., 2013). Each line was repeated thrice, and the leaves from 3 plants were selected for each repetition. The thickness of the leaf tissue slice was 10 μm. Safranine-fast green staining was performed, and the tissue was observed under an optical microscope (OLYMPUS BX43F). The leaf PT, sponge tissue (ST) thickness, PT cell density, and PT cell density were determined using Image J (Schneider et al., 2012). Statistics were gathered from 3 slices per plant.

The calculation formula used was as follows:


$$\frac{PT}{ST}=\frac{PT\;thickness}{ST\;thickness\;}\;\times \;100\%$$

PT cell density is the number of cells per unit length (100 μm).

### Determination of endogenous IAA content in transgenic birch

The RE, OE, and WT lines showing consistent growth were selected, and the apical buds and one leaf were respectively picked. Twelve ramets were collected from each line. Endogenous phytohormones were extracted from the samples using a mixture of isopropanol, water, and hydrochloric acid. The content of endogenous phytohormones in plants was determined using the UPLC-MS/MS platform composed of the Agilent 1290 High-performance Liquid Chromatograph and Absie QTRAP 6500 + mass spectrometer. Cavens Detection Technology Co., Ltd. provided both auxin standards and antibodies. To maintain a high degree of precision, the samples were run thrice.

### Tissue localization of endogenous IAA in birch leaf suppression expression by *BpPIN3* and *GUS* enzyme activity

The leaves of tissue culture seedlings from the *BpPIN3* RE lines were used. The leaves pre-cultured for 2–3 days were inoculated with EHA105 (proDR5::*GUS*) bacterial solution at OD600 of approximately 0.5 for 10–20 min. Under the screening condition of 50 mg/L hygromycin B (Hyg), the RE (proDR5:: *GUS*)-resistant calli were obtained. The resistant calli were inoculated into the differentiation medium containing 50 mg/L Hyg to induce the formation of adventitious buds, and multiple subculture seedlings were obtained. A rooting culture was performed on the solid medium containing WPM + 0.4 mg/L IBA. After 10 days, *GUS* staining was performed on the whole seedlings (Jefferson et al., 1987). After *GUS* staining, the leaves were cut and photographed using a stereomicroscope. The leaves of RE and WT lines after *GUS* staining were sliced by using the conventional paraffin method (Liu et al., 2013). Each line was repeated thrice, and the leaves of 3 individual plants were selected for each repetition. The thickness of the slice was 10 μm, and 0.02% ruthenium red was used for staining. After neutral resin sealing, the optical microscope (OLYMPUS BX43F) was used for observation and photography.

The leaf margin and the main vein region of the RE and WT lines were used as materials. Quantitative measurements of the *GUS* activity were conducted using the Plant *GUS* ELISA Kit (Cat. No. JN0; Jining Shiye, Shanghai, China).

### RNA-seq and differential gene expression analysis

RNA was extracted from the mature leaves of the RE, OE, and WT lines and treated with DNaseI. Three biological replicates per line were established. Each biological replicate had 6 individual leaves. RNA sequencing was performed by the BGI Group on the DNBSEQ-T7 sequencing platform. Bowtie2 was used for a comparative analysis of the birch reference genome after the removal of low-quality reads (http://bowtie-bio.sourceforge.net/bowtie2/index.shtml). Based on the RNA-seq data, DEGs were calculated using the maximum expectation algorithm (Tusher et al., 2001). In addition, the DEGs were annotated based on the following databases: NCBI/Nr (http://www.ncbi.nlm.nih.gov), NCBINt (http://www.ncbi.nlm.nih.gov), Swiss-Prot (http://www.uniprot.org/keywords/), and KEGG pathway database (http://www.genome.jp/kegg). The expression level of each gene was calculated using FPKM (Florea et al., 2013). EdgeR was used to define the DEGs between each two-sample comparison, with the thresholds set as follows: the false discovery rate (FDR) ≤ 0.01, Log2FC ≥ 1, and Log2FPKM ≥ 1 (Edge R package; version 3.28.1). The target genes were first annotated in the GO database (http://www.geneontology.org/) and then classified into the corresponding functional categories to evaluate their biological functions. In addition, the KEGG pathway enrichment analysis was performed using the KEGG pathway database (http://www.genome.jp/kegg).

### Statistical analysis

One-way or two-way analysis of variance was used for statistical analysis. Tukey’s method was used for multiple comparisons. Data are expressed as the mean ± standard deviation (SD). The difference is denoted in four levels, significant *p< 0.05, highly significant **p< 0.01, extremely significant ***p< 0.001, and highly extremely significant ****p< 0.0001.TBtools (Chen et al., 2020), SPSS22.0, and GraphPad Prism (GraphPad Prism 8; GraphPad) were used to analyze and plot the data.

## Conclusion

*BpPIN3* is involved in leaf polarity formation in birch. The decrease in *BpPIN3* expression led to an increase in the auxin response-related gene expression in birch leaves, which affected the polar transport of auxin, resulting in a difference in auxin concentration in the leaves. The excessive auxin at the leaf margin modified the structure of PT cells, leading to upward curling at the leaf margin.

## Data availability statement

The original contributions presented in the study are publicly available. This data can be found here: NCBI, PRJNA885271.

## Author contributions

KC, G-FL and JJ designed the research, KC and CQ conducted the experiments, KC conducted the data analysis and wrote the manuscript, KC, WW and C-RG performed transcriptome data analysis, X-YZ, Q-BY, CY and JJ revised the manuscript, X-YZ participated some partial index measurement. Q-BY has carried on the experiment instruction and the experimental design consummation. All authors contributed to the article and approved the submitted version.

## Funding

This research was supported by the National Key R&D Program of China during the 14th Five-year Plan Period (2021YFD2200103) and Heilongjiang Touyan Innovation Team Program (Tree Genetics and Breeding Innovation Team).

## Acknowledgments

Thanks for the technical platform and support provided by the State Key Laboratory of Tree Genetics and Breeding, Northeast Forestry University.

## Conflict of interest

The authors declare that the research was conducted in the absence of any commercial or financial relationships that could be construed as a potential conflict of interest.

## Publisher’s note

All claims expressed in this article are solely those of the authors and do not necessarily represent those of their affiliated organizations, or those of the publisher, the editors and the reviewers. Any product that may be evaluated in this article, or claim that may be made by its manufacturer, is not guaranteed or endorsed by the publisher.
