# Supplementary material for: Molecular mechanism of leaf adaxial upward curling caused by BpPIN3 suppression in Betula pendula
Source: Front Plant Sci. 2022 Dec 1;13:1060228. doi: 10.3389/fpls.2022.1060228 (PMC9751824; doi:10.3389/fpls.2022.1060228)
Supplement: Supplementary file 1 [file DataSheet_1.docx]

Molecular Mechanism of Leaf Adaxial Upward Curling Caused by *BpPIN3* Suppression in *Betula pendula*

Kun Chen^1†^, Chang Qu^1†^, Xiao-yue Zhang^1^, Wei Wang^1^, Chen-rui Gu^1^, Gui-feng Liu^1^, Qi-bin Yu^2^, Chuanping Yang^1*^, Jing Jiang^1*^

^1^ State Key Laboratory of Tree Genetics and Breeding, Northeast Forestry University, 26 Hexing Road, Harbin 150040, China

^2^ Citrus Research and Education Center, University of Florida, Lake Alfred, FL 33850, United States

*** Correspondence:**Corresponding Author
[yangcp@nefu.edu.cn](mailto:yangcp@nefu.edu.cn);[jiangjing@nefu.edu.cn](mailto:jiangjing@nefu.edu.cn)

^†^These authors share first authorship

# Supplementary Figures


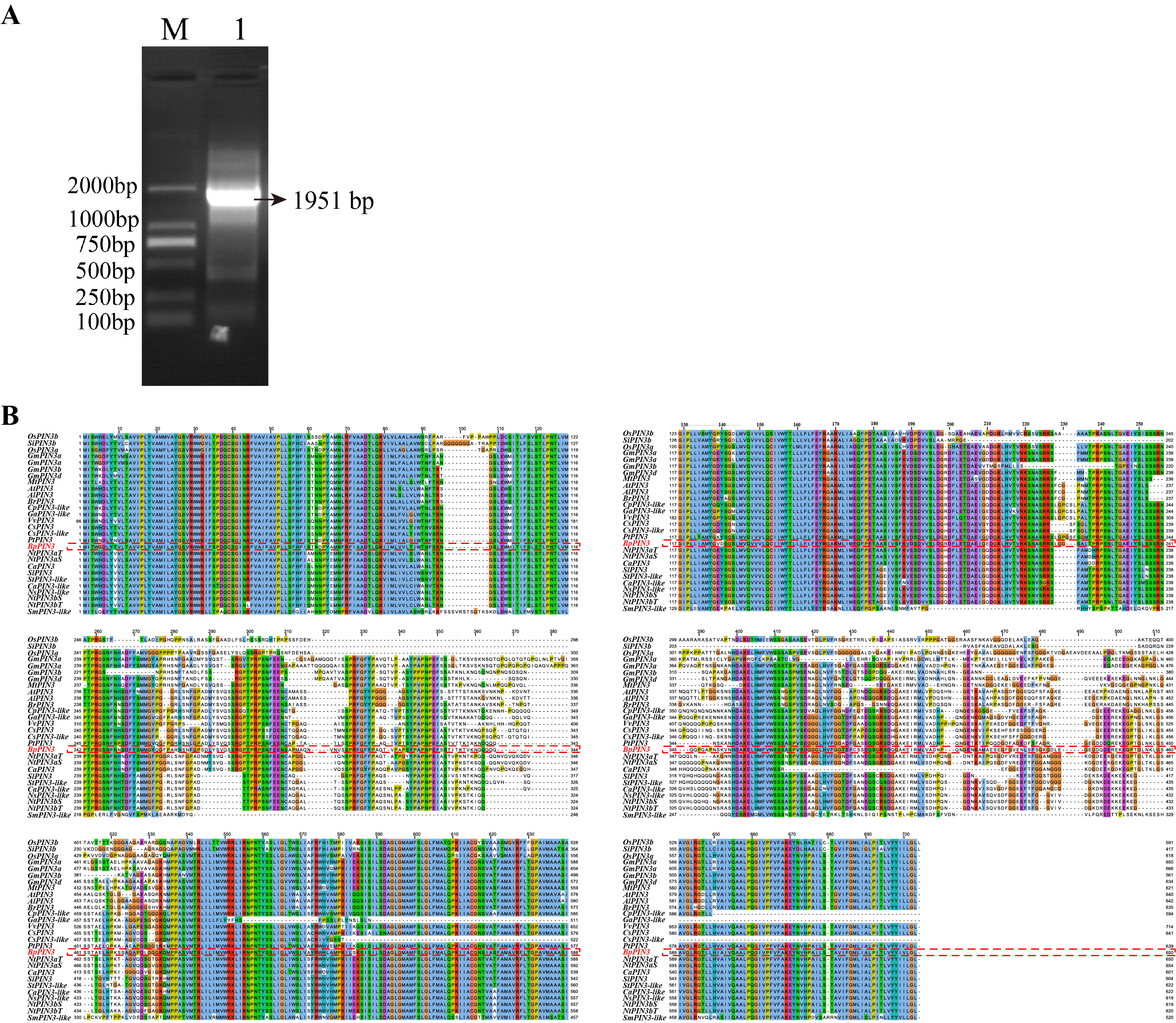


**Supplementary Figure 1.** *BpPIN3* Amplified and sequence alignment

A. *BpPIN3* Amplified Electrophoresis Map M: DNA Marker, DL 2000; 1: Full length of *BpPIN3* gene. B. *BpPIN3* amino acid sequence alignment with other species *PIN3*

.
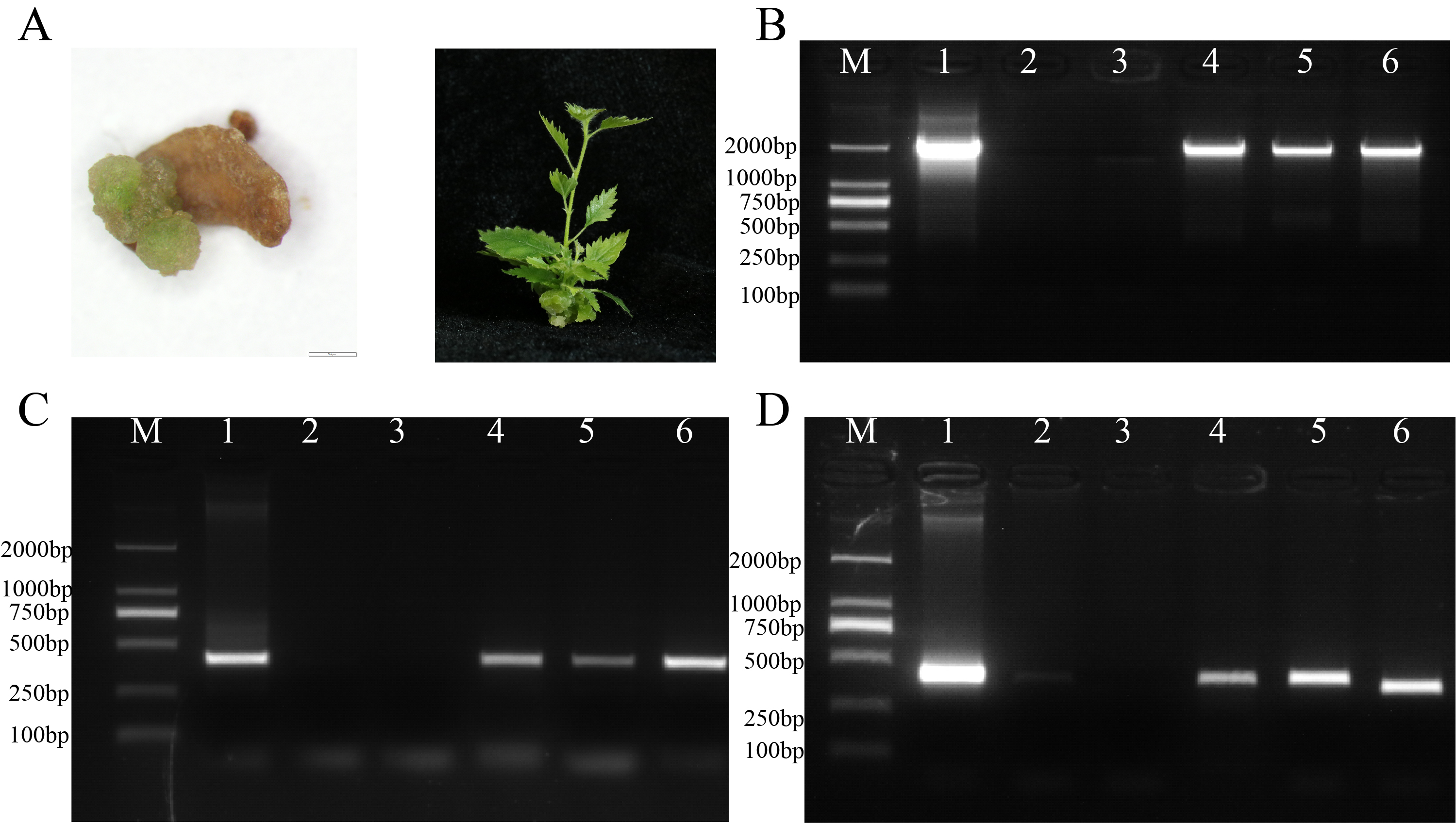


**Supplementary Figure 2.** Acquisition and identification of transgenic birch *BpPIN3*

A. Tissue culture seedlings ( The left is a resistant callus, the right is subculture seedlings ); B. PCR results of OE strain M: DNA Marker, DL 2000; 1: Positive plasmid control 2: Water control ; 3:WT line; 4-6. OE1-OE3 lines; C. RE strain forward target sequence PCR amplification; D. RE strain reverse complementary sequence PCR amplification M: DNA Marker, DL 2000; 1:Positive plasmid control; 2: Water control; 3: WT birch; 4-6: RE1-RE3





**Supplementary Figure 3 BpPINs expression in RE and WT**

**Relative transcript levels of BpPINs were analyzed by qRT-PCR in leaf of RE1-3 and WT seedlings. Different letters indicate statistically signiﬁcant differences by ANOVA, P≤0.05. Error bars are ±SD, n=3**


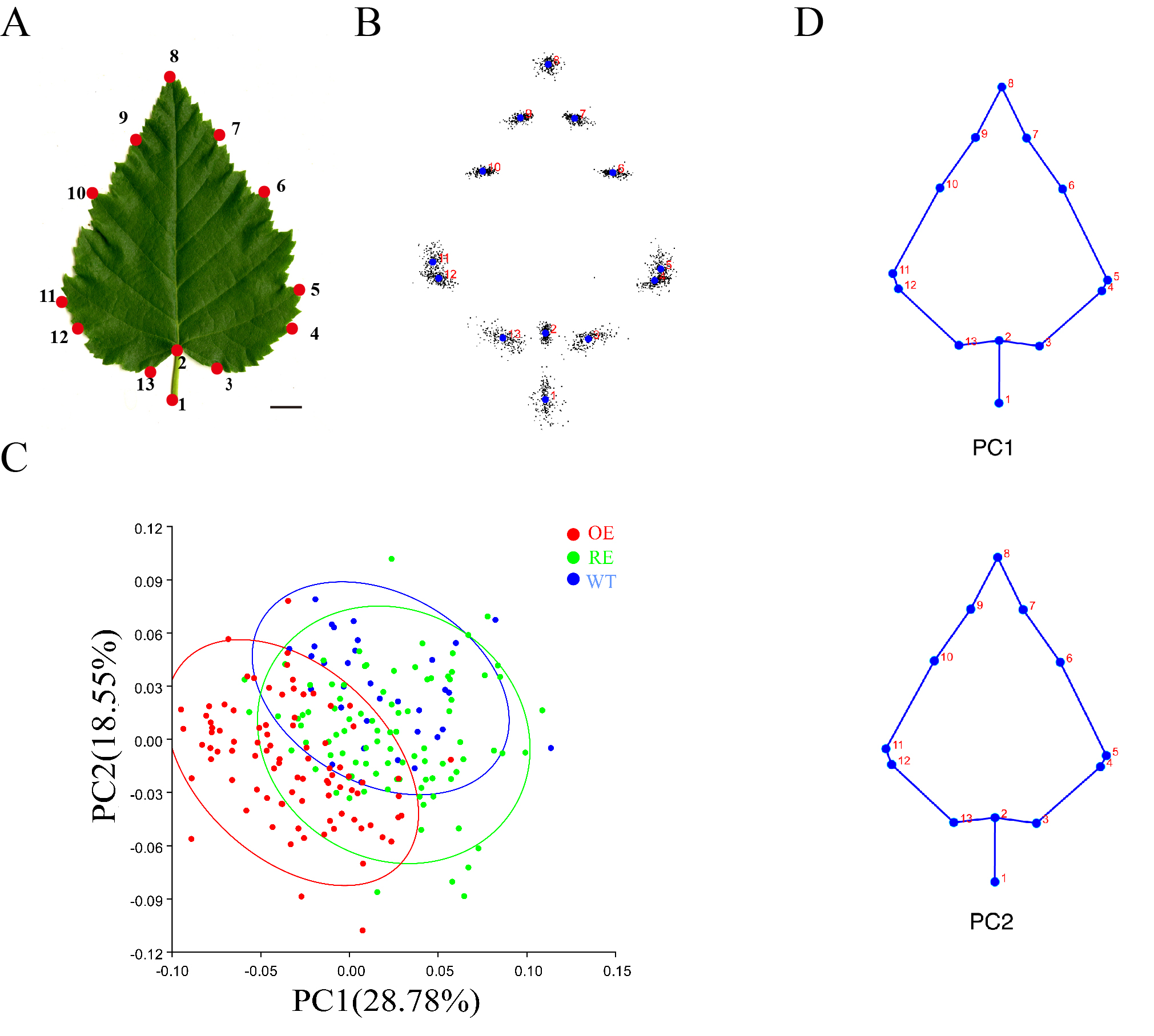


**Supplementary Figure 4.** Principal component analysis of leaves of RE, OE, and WT lines

A. Thirteen landmarks for leaf analysis. Bar = 1cm ; B. Covariance matrix after Procrustes analysis; C. PCA analysis scatter plot; D. PC1 and PC2 wireframes.


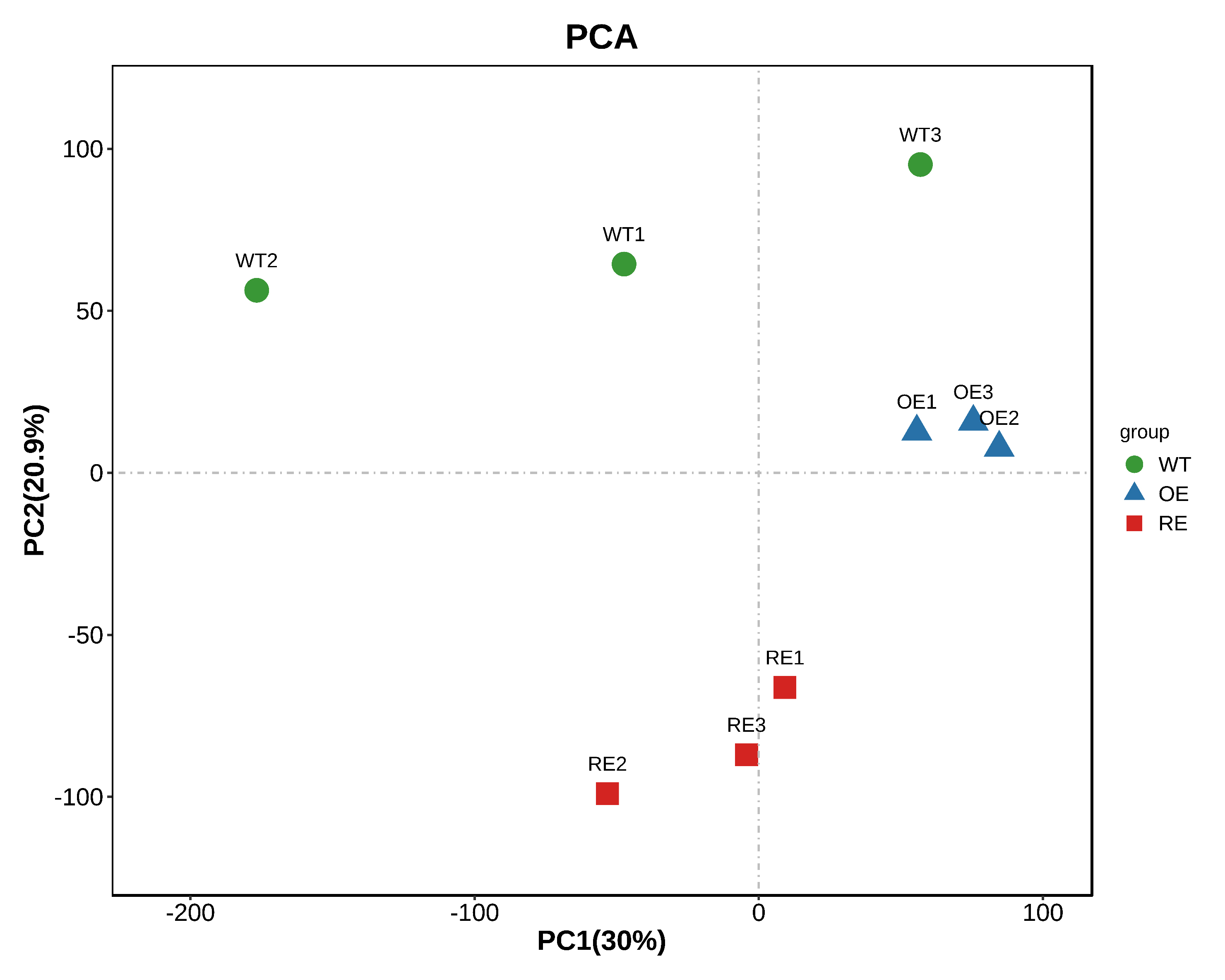


**Supplementary Figure 5.** Transcriptome data principal component analysis (PCA).


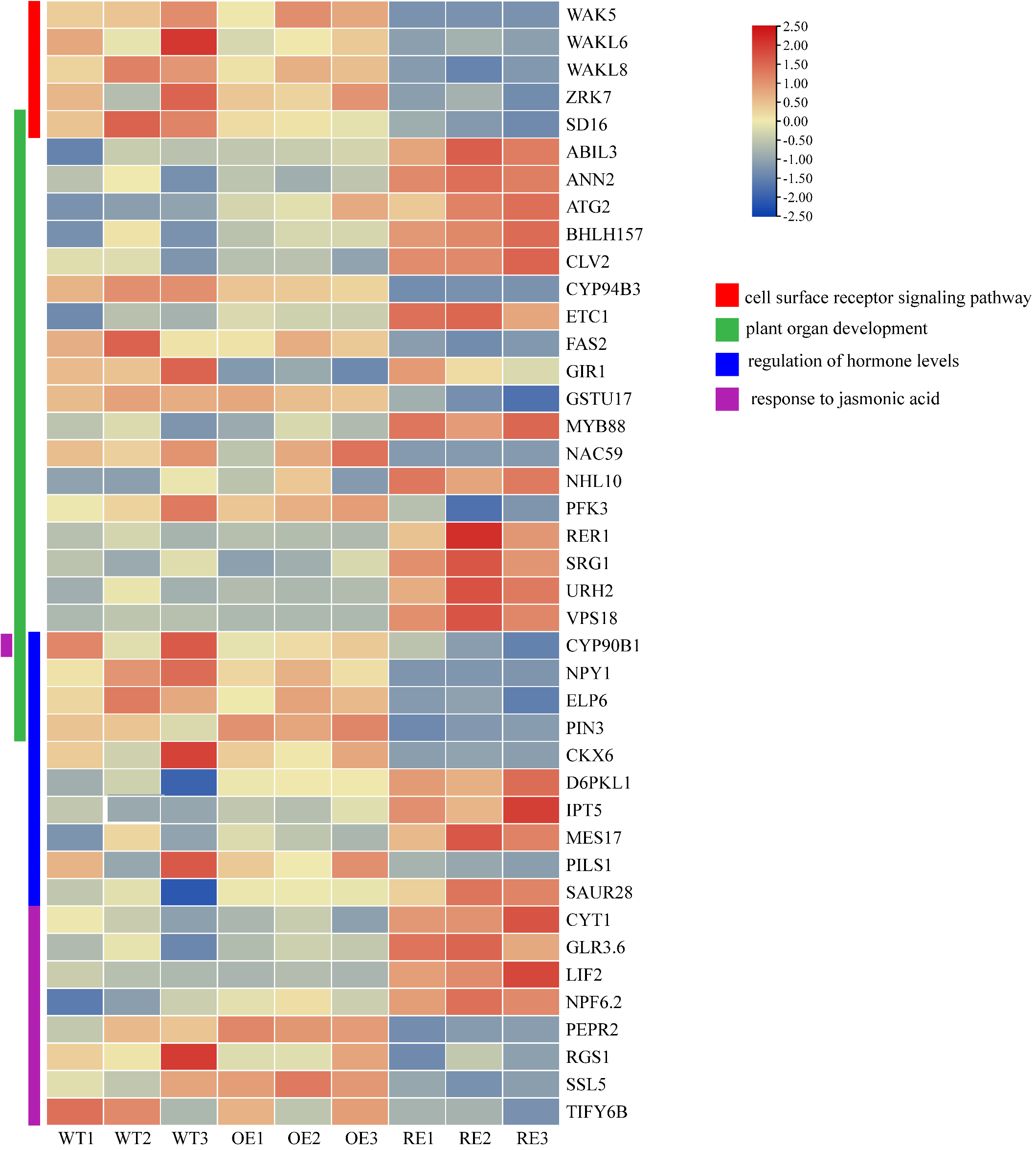


**Supplementary Figure 6.** Heatmap analysis of the transcriptome

A gene heat map of plant development related pathways is shown after GO enrichment analysis. Every gene is indicated in a row, and each column contains data from a separate sample.


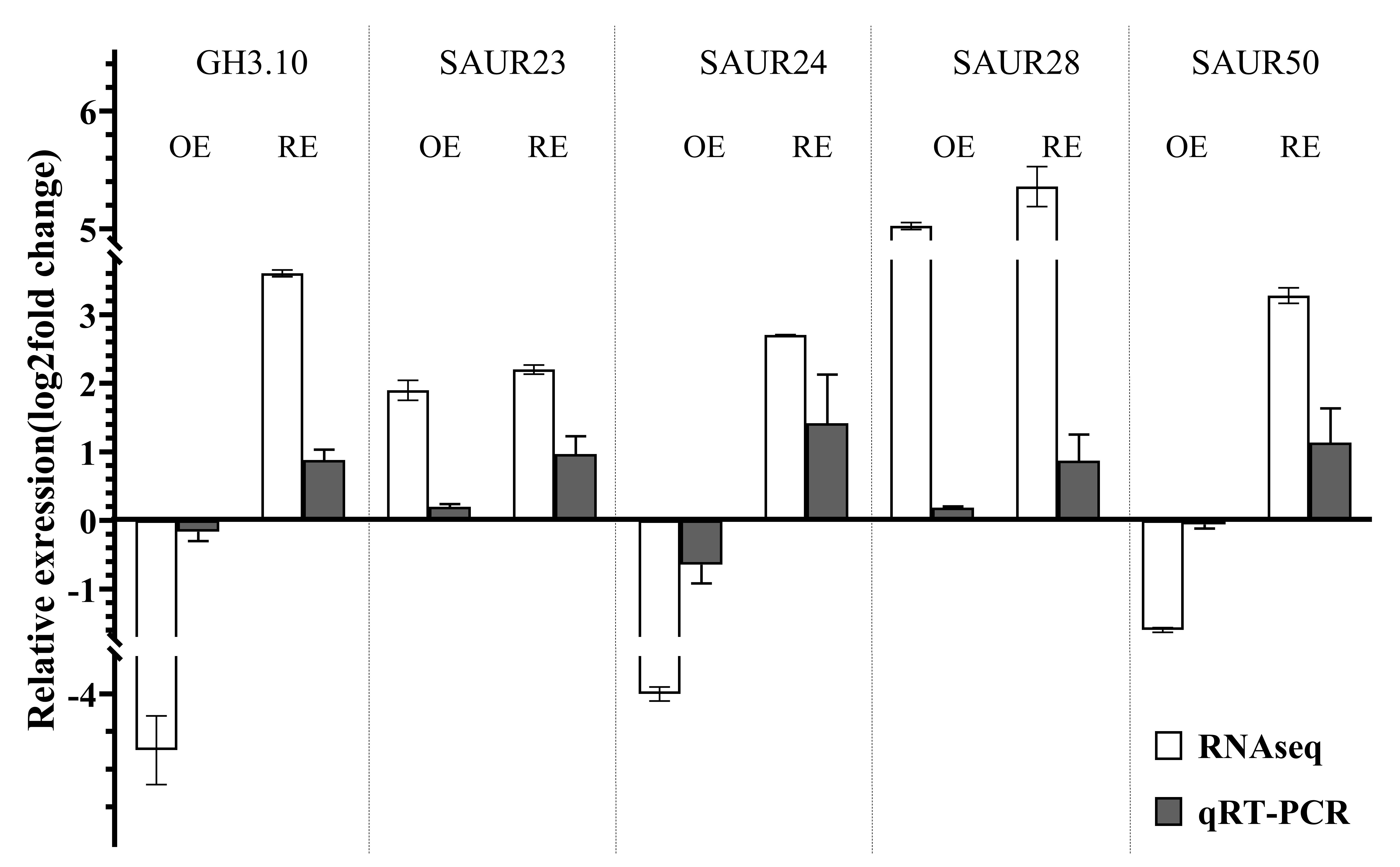


**Supplementary Figure 7 qRTPCR validation of the transcriptome data.**

Student’s t-test. Results are shown as mean expression ± standard deviation (SD) of three independent experiments.


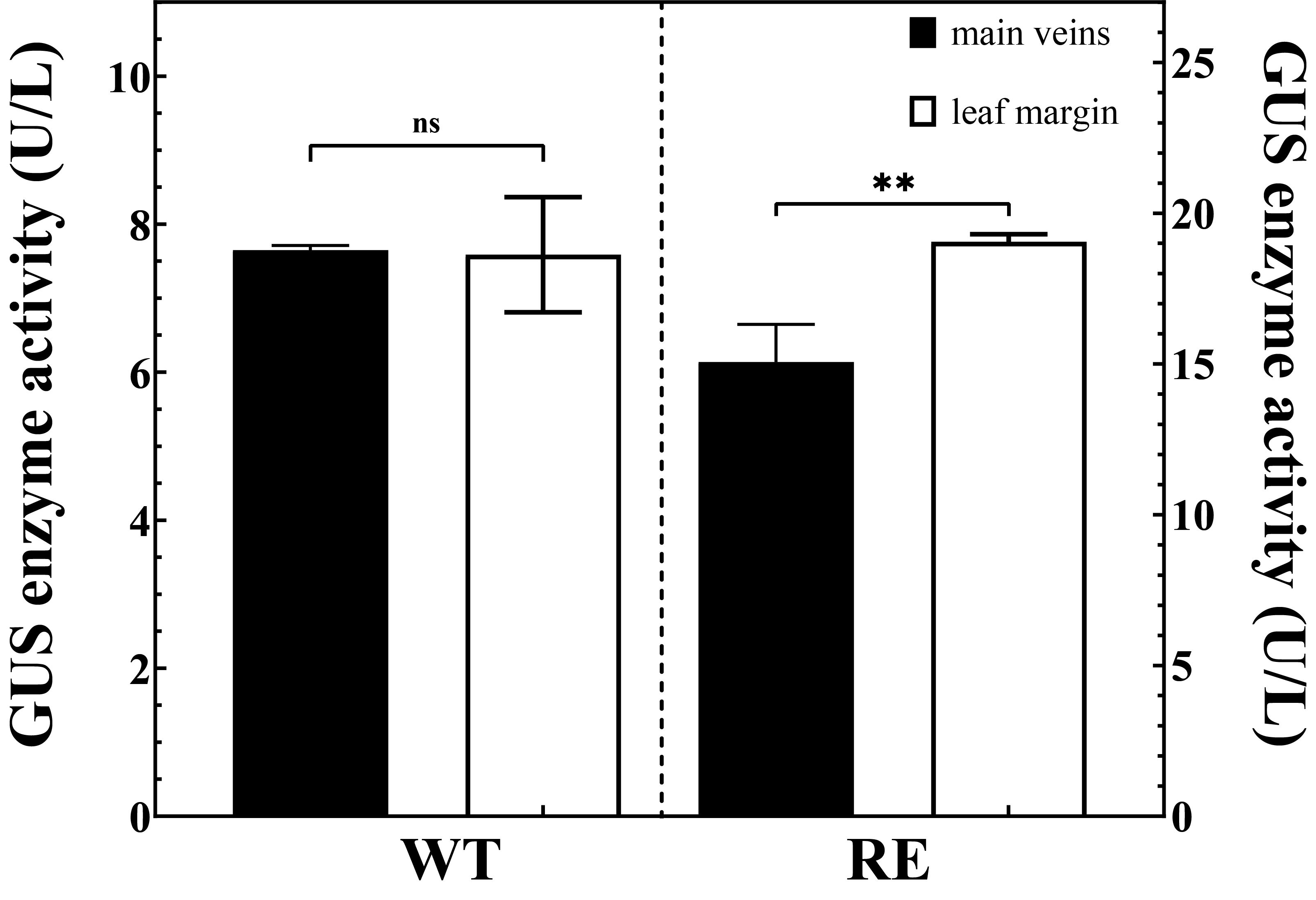


**Supplementary Figure 8** **GUS activity in leaf margin and main vein of transgenic plants.**

WT ( proDR5:: GUS ),RE ( proDR5:: GUS ).Student’s t-test, *P<0.05, **P<0.01. Results are shown as mean expression ± standard deviation (SD) of three independent experiments.



**Supplementary Figure 9.** Pearsons correlation analysis of relative expression of *GUS* and *BpPIN3* gene in each region of RE strain leaves





**Supplementary Figure 10.** Relative expression of *GH3.10* and SAURs genes in different regions of RE lines leaves. Errorbars indicate SD (mean±SD), n=3

# Supplementary Tables

**Supplementary Table S1** Construction of over-expression vector primer sequence

| Primer name | primer sequences（5′-3′） |
| --- | --- |
| *BpPIN3-F* | CGCGGATCCATGATCACCTGGGGAGACCTGTAC |
| *BpPIN3-R* | CGGGGTACCTCACAATCCCAGAACGATGTAGTAGACAAG |

**Supplementary Table S2** Leaf index determination of transgenic plants

Errorbars indicate SD (mean±SD), n=30.Different letters denote statistically significant differences after one-way ANOVA.

| leaf index | petiole length (Pl)（cm） | leaf length (Ll)（cm） | leaf width (Lw)（cm） | leaf area (La)（cm^2^） | leaf perimeter (LP)（cm） | LI/PL | | LI/LW |
| --- | --- | --- | --- | --- | --- | --- | --- | --- |
| WT | 1.85±0.26ab | 8.36±0.58a | 6.7±0.54a | 37.29±5.81a | 41.46±2.96a | 4.62±0.75a | 1.25±0.05a | |
| OE1 | 1.53±0.26c | 6.48±0.57c | 5.88±0.57b | 28.86±5.77b | 37.22±4.62b | 4.35±078ab | 1.1±0.06d | |
| OE2 | 1.52±0.25c | 6.67±0.49bc | 5.81±0.58bc | 28.71±5.81b | 37.47±4.99b | 4.52±0.88a | 1.15±0.07c | |
| OE3 | 1.49±0.31c | 6.59±0.75bc | 5.66±0.68bcd | 28.13±6.78b | 37.77±5.23b | 4.6±0.93a | 1.17±0.06c | |
| RE1 | 1.71±0.28b | 6.61±0.46bc | 5.36±0.65d | 24.94±5.4c | 31.5±5.97c | 4±0.86bc | 1.24±0.1ab | |
| RE2 | 1.77±0.33ab | 6.66±0.6bc | 5.51±0.52cd | 26.03±4.55bc | 33.47±3.06c | 3.91±0.98c | 1.21±0.07b | |
| RE3 | 1.90±0.26a | 6.82±0.53b | 5.69±0.62bc | 27.3±5.14bc | 33.34±3.12c | 3.66±0.64c | 1.21±0.11b | |

**Supplementary Table S3 Seedling height of transgenic plants**

Errorbars indicate SD (mean±SD), n=9.Different letters denote statistically significant differences after one-way ANOVA.

| line | seedling height(cm) |
| --- | --- |
| WT | 109.4±8.49a |
| OE1 | 64.38±15.96c |
| OE2 | 89.3±10.44b |
| OE3 | 104.77±11.94a |
| RE1 | 114.41±21.47a |
| RE2 | 112.33±18.23a |
| RE3 | 100.86±17.55ab |

**Supplementary Table S4** *BpPIN3*-RNAi vector construction primer sequence

|  | Primer name | restriction enzyme cutting site | primer sequences（5′-3′） |
| --- | --- | --- | --- |
| targeting DNA | P3Ri1 | NcoI | CATGCCATGGAACACCTATTCCAGCCTCATTGGC |
|  | P3Ri2 | AscI | TTGGCGCGCCATAGCAAAAGAAGCGATTGAGTTCCC |
| Targeted DNA reverse complementary sequence | P3Ri3 | XBaI | GCTCTAGAAACACCTATTCCAGCCTCATTGGC |
|  | P3Ri4 | BamHI | CGCGGATCCATAGCAAAAGAAGCGATTGAGTTCCC |

**Supplementary Table S5** Formula of birch tissue culture medium

| Name of medium | culture medium formula |
| --- | --- |
| Co-culture medium | WPM＋2.0mg/L 6-BA＋0.2 mg/L NAA |
| selective medium | WPM＋2.0mg/L 6-BA＋0.2 mg/L NAA |
| differential medium | WPM＋0.8mg/L 6-BA＋0.1mg/L NAA +0.5mg/LGA_3_ |
| Subculture medium | WPM＋0.8mg/L 6-BA＋0.1mg/L NAA |
| rooting culture medium | WPM＋0.4mg/L IBA |
| Screening agent concentration | Overexpression : 50 mg / L kan ; reduced expression : 5 mg / L glyphosate |

**Supplementary Table S6** Gene ID and primers used for qRT-PCR

| Gene name | Forward primer（5′-3′） | Reverse primer（5′-3′） |
| --- | --- | --- |
| Bpev01.c2175.g0002(*GH3.10*) | ACATCAGAGAAGGCAACCTCA | ATCAAACCACCCCAATTCAGA |
| Bpev01.c1577.g0016(*SAUR23*) | ATAGATGTTCCAAAAGGCCACC | ATTCTTCCTCAGCCTTGCCTA |
| Bpev01.c1577.g0018(*SAUR24*) | ATGTACCAAGAGGCTTCCTT | CCACCCGTAGGATGATTAAACCC |
| Bpev01.c1577.g0015(*SAUR28*) | ATTCTCCGTCGATCAAACTCC | ATACTGGAACTACGAATCGCTT |
| Bpev01.c1638.g0007(*SAUR50*) | GCAGTTCTCAAGCAAATCCTG | TGTCTGCTTCTCTTTTCCCCAA |
| Bpev01.c1162.g0001(*PIN3*) | CCTCTCCTTCCACTTCATCTCTACC | GGACGACGAGCATGATAATCTTCTG |
| Bpev01.c0147.g0002(*PIN1*) | CATCAACCACTCCCAGACCC | GTTCGAGTTCCTCCCACCAG |
| Bpev01.c0606.g0011(*PIN2*) | GAAGGAGAGTGGTGGTGCAA | TTTCGTGTTGAAGCGCAGC |
| Bpev01.c1418.g0011(*PIN4*) | CCTCCACAGCAGACAATCACT | CTGTGTTCCCGAATGCGATG |
| Bpev01.c0281.g0015(*PIN5*) | CCTCTGCTGTCTTTCCAAGTGA | GGCAGTGTTGACAGGGAGAT |
| Bpev01.c1399.g0005(*PIN6*) | TCCGGTCTCTCACTCACTCC | CATTGCTCGCGTTCTTACCG |
| *GUS* | CACCTGGGTGGACGATATCAC | CATCAATCACCACGATGCCATG |
| *18S* | GAGGTAGCTTCGGGCGCAACT | GCAGGTTAGCGAAATGCGATAC |
